# Evolution of the Metallographic Structure of Additively Manufactured Ti-6Al-4V and Ti-6Al-7Nb Titanium Alloys

**DOI:** 10.3390/ma19010080

**Published:** 2025-12-25

**Authors:** Dorota Laskowska, Błażej Bałasz, Łukasz Żurawski

**Affiliations:** Faculty of Mechanical Engineering and Energy, Koszalin University of Technology, Śniadeckich 2, 75-453 Koszalin, Poland; lukasz.zurawski@tu.koszalin.pl

**Keywords:** additive manufacturing, titan-base alloys, heat treatment, microstructural transformation, mechanical properties

## Abstract

The aim of this study was to evaluate structural changes and their impact on the functional properties of Ti-6Al-4V and Ti-6Al-7Nb titanium alloys produced by L-PBF. In the as-built condition, these alloys, despite high strength due to the presence of metastable α’ martensite, exhibit limited ductility. The samples were subjected to heat treatment at 850–1000 °C for 1 h, followed by aging at 500 °C for 4 h in an argon atmosphere. Analysis revealed a gradual microstructural transformation from the columnar structure characteristic of L-PBF to an equilibrium Widmanstätten microstructure. As a result of the decomposition of martensite and the formation of an α + β phase mixture, changes in microhardness and mechanical properties were observed. After heat treatment, the microhardness decreased by 15% for Ti-6Al-4V (from 427 ± 1 HV to 362 ± 25 HV) and by 12% for Ti-6Al-7Nb (from 408 ± 6 HV to 359 ± 15 HV). The Ti-6Al-7Nb alloy exhibited higher maximum elongation (7.7 ± 1.1%) than Ti-6Al-4V (4.8 ± 0.5%) due to a greater fraction of the β phase. The results highlight the critical role of the controlled α′→α + β transformation in tailoring the properties of titanium alloys and provide a basis for optimizing manufacturing processes for medical and aerospace components.

## 1. Introduction

Titanium and its alloys are extensively utilized in dental medicine, orthopedics, and implantology due to their exceptional corrosion resistance, highly specific mechanical properties, and biocompatibility. In response to the growing demand for components with highly customized geometries and tailored properties, additive manufacturing technologies—particularly laser powder bed fusion (L-PBF)—have experienced significant advancements in recent years. L-PBF operates by selectively melting a powder bed in accordance with a CAD model using a high-energy laser. Comprehensive details regarding the technology are provided in [1,2,3].

Among titanium alloys, Ti-6Al-4V is the most commonly employed in medical applications. However, it is anticipated that non-vanadium alloys may see wider application due to the reported cytotoxicity of vanadium ions [4,5]. Studies conducted by Costa et al. [6] demonstrated that the continuous exposition for V ions and their accumulation in surrounding tissues can induce cytotoxic effects, raising concerns regarding long-term biocompatibility—particularly for implants susceptible to corrosion or wear. Furthermore, research has shown that Ti-6Al-7Nb exhibits superior corrosion resistance compared to Ti-6Al-4V [7,8]. Both alloys are α + β dual-phase alloys, in which aluminum acts as the α-phase stabilizer, while vanadium and niobium serve as the β-phase stabilizers, respectively [9].

Several factors influence the properties of titanium alloys, among which microstructure is one of the most significant. It is primarily governed by the alloy’s chemical composition and the manufacturing process. The specific characteristics of the L-PBF process—particularly the high temperature of the molten pool and the rapid solidification times—lead dual-phase titanium alloys to develop a microstructure composed of non-equilibrium acicular α′ martensite alongside large columnar β grains. Such a microstructure leads to high hardness and tensile strength but low ductility [10,11]. According to ISO 5832-3:2021 [12], Ti-6Al-4V intended for surgical implant production should achieve a minimum elongation of 10%. However, Yan et al. [13], Vilaro et al. [14], and Gong et al. [15] reported elongation values of 4.4 ± 0.7%, 1.7 ± 0.3%, and 5.4 ± 3.8%, respectively. Studies by Laskowska et al. [16] demonstrated that Ti-6Al-7Nb produced via L-PBT exhibits greater elongation compared to Ti-6Al-4V. The maximum elongation recorded for Ti-6Al-7Nb was 7.6 ± 0.9%, roughly four times higher than that observed for Ti-6Al-4V (2.2 ± 0.2%). Xu et al. [17] reported an elongation of 9.7 ± 0.3% for the Ti-6Al-7Nb alloy.

To enhance the reproducibility and stability of the functional properties of titanium components produced via additive manufacturing, appropriate heat treatment plays a crucial role. Heat treatment allows for controlled modification of the microstructure [18,19,20], reduction in residual stresses [21,22], and tailoring of mechanical properties to meet application-specific requirements [23,24,25]. The selection of heat treatment parameters is a complex issue, depending both on the employed additive manufacturing technology and the specific characteristics of the titanium alloy. Key parameters include the annealing temperature and duration [26,27] as well as the cooling rate [28].

Many authors highlight that the selection of an appropriate heat treatment strategy represents a trade-off, where improvements in ductility and reduction in residual stresses may be accompanied by a decrease in hardness and tensile strength [29,30,31]. Zhao et al. [32] demonstrated that heat treatment of Ti-6Al-4V titanium alloy within the temperature range of 750–950 °C leads to the decomposition of α′ martensite and the formation of an α + β structure, resulting in a significant increase in ductility with an acceptable reduction in tensile strength. Studies conducted by Cao et al. [33] confirmed these findings while also indicating that further temperature increases lead to excessive grain growth and a deterioration in the homogeneity of mechanical properties.

Despite extensive studies on Ti-6Al-4V and Ti-6Al-7Nb alloys processed via additive manufacturing, most reports have focused on individual alloys or on different processing and heat treatment conditions. A systematic, side-by-side comparison of these two alloys subjected to identical L-PBF parameters and thermal cycles has not yet been reported. Such a direct comparison is crucial to elucidate the influence of Nb substitution on phase stability, microstructure evolution, and mechanical behavior under the same processing conditions. Therefore, the present study aims to fill this research gap by providing a comprehensive analysis of both alloys, highlighting the effects of heat treatment on their microstructure and mechanical properties, and offering guidance for application-specific optimization.

## 2. Materials and Methods

### 2.1. Construction Material

The samples were fabricated using commercially available titanium alloy powders: Ti-6Al-4V (3D Systems, Rock Hill, SC, USA) and Ti-6Al-7Nb (SLM Solution Group AG, Lübeck, Germany). The chemical composition of each powder, based on the manufacturers’ certificates, is summarized in Table 1.

### 2.2. Samples Fabrications

Samples for this study were fabricated using L-PBF technology with an ORLAS CREATOR^®^ system (O. R. Lasertechnologie GmbH, Dieburg, Germany) equipped with a ytterbium fiber laser, featuring a beam spot diameter of 40 μm, a maximum power of 250 W, and a wavelength of 1070 nm. The build chamber was filled with argon, maintaining a residual oxygen level below 0.1%. Process parameters were selected based on the study by Laskowska et al. [16], and the set of parameters applied is summarized in Table 2.

### 2.3. Heat Treatment Procedure

The samples were subjected to heat treatment using a Nabertherm tube furnace (Nabertherm, Lilienthal, Germany) under a high-purity argon atmosphere. Four heat treatment procedures were carried out, with their parameters presented in Table 3. The samples were annealed at 850 °C, 900 °C, 950 °C, or 1000 °C for 1 h, followed by aging at 500 °C for 4 h. All samples were subsequently slow-cooled inside the furnace to room temperature with a cooling rate of 200 °C/1 h.

For each heat treatment strategy, three cubic samples (10 × 10 × 10 mm) and three tensile test specimens (with dimensions compliant with the ISO 6892-1:2020-05 standard [34]) were fabricated. The tensile bars were built in a vertical orientation relative to the build platform.

### 2.4. Material Characteristics

Post-processing of the samples included mechanical removal of the support structures followed by ultrasonic cleaning in distilled water for 10 min. The characterization of the samples was carried out according to the procedure described by Laskowska et al. [16].

#### 2.4.1. Relative Density

Relative density measurements were performed using a Mettler Toledo XS105 hydrostatic balance (Mettler Toledo, Columbus, OH, USA). The relative density of the samples was measured both before and after heat treatment. The samples were divided into four groups, with three samples in each group. For each sample, three measurements were conducted.

#### 2.4.2. Surface Topography

Surface topography was analyzed using an InfiniteFocus G6 3D optical microscope (Alicona Imaging GmbH, Graz, Austria). Measurements were conducted both before and after heat treatment. The samples were divided into four groups, with three samples in each group. An 800 WD17 objective (Alicona Imaging GmbH, Graz, Austria) was used, enabling data acquisition from the entire top surface, corresponding to an area of 10 mm × 10 mm. One measurement was performed for each sample.

The acquired data were analyzed using TalyMap Platinum v7.4 software (Taylor Hobson, Leicester, UK). First, a 6 mm × 6 mm region was extracted from the original measurement area to avoid edge-related irregularities in the surface roughness evaluation. Surface roughness was assessed using the arithmetic mean height (Sa) and maximum height (Sz) parameters, in accordance with ISO 25178-2:2021 [35].

#### 2.4.3. Microhardness

The samples for microhardness testing were sliced using a water-cooled diamond blade. The obtained cross-sections were embedded in DuroFast epoxy resin (Struers, Copenhagen, Denmark), providing a suitable shape for subsequent preparation using a LaboPol-30 grinding and polishing machine (Struers, Copenhagen, Denmark). The samples were ground and polished following the preparation protocol developed by Struers.

Microhardness measurements were performed using a FISCHERSCOPE HM2000 microhardness tester (Helmut Fischer GmbH, Sindelfingen, Germany). To analyze local hardness values, the sample cross-section was divided into five regions, with four measurements performed in each region.

#### 2.4.4. Microstructure

The material microstructure was initially analyzed using metallographic specimens (prepared according to the methodology described in Section 2.4.3) etched with Kroll’s reagent (Chempur, Piekary Śląskie, Poland). The etching time was 15 s. Images were captured using a NIKON MA200 optical microscope (Nikon, Minato, Tokyo, Japan).

Metallographic specimens were analyzed for phase composition using an Empyrean X-ray diffractometer (Malvern Panalytical Ltd., Malvern, UK) equipped with a Cu-Kα (λ = 1.5406 Å) radiation source. The study was carried out in Bragg–Brentano geometry over a 2θ range of 30–80°.

The crystallographic orientation was investigated using electron backscatter diffraction (EBSD) with a FEI Quanta 3D FEG scanning electron microscope integrated with an EDAX Trident system (Thermo Fisher Scientific, Waltham, MA, USA). The analyses were conducted at the accredited laboratory of the Institute of Metallurgy and Materials Engineering, Polish Academy of Sciences. Measurements were performed on three selected samples: S0–as-built, S3–950, and S4–1000. Orientation mapping was carried out using an accelerating voltage of 20 kV and an electron beam current of 16 nA. The orientation map was collected over an area of 193 µm × 193 µm with a step size of 0.1 µm. The working distance between the electron beam focus point and the objective pole piece was set to WD = 10 mm.

TLS OIM Analysis 8.0 software (Ametek EDAX, Mahwah, NJ, USA) was used to analyze the orientation distribution and determine the average grain size. A grain was defined as a region comprising at least three points with the same orientation, separated from neighboring grains by a boundary with a misorientation angle greater than 10°. Raw measurement data were re-indexed using the Neighbor CI Correlation method, with a confidence index (CI) threshold of 0.2. Data points with a CI value below 0.2 were excluded from the analysis. Table 4 presents data on the number of identified grains.

#### 2.4.5. Mechanical Properties

To assess the influence of heat treatment on the mechanical properties of the studied alloys, uniaxial tensile tests were performed using a Zwick Z400E universal testing machine with a macroXtens extensometer (ZwickRoell GmbH, Ulm, Germany). Tests were carried out at room temperature in line with ISO 6892-1:2020-05 [34], with five specimens tested per series. All tensile tests were conducted parallel to the build direction. Ultimate tensile strength (Rm), yield strength (Rp0.2), and Young’s modulus (E) were extracted from the stress–strain curves using testXpert III v1.4 software (ZwickRoell GmbH, Ulm, Germany).

## 3. Results and Discussion

### 3.1. Relative Density

Relative density is widely used as a key metric for evaluating the quality of additively manufactured components and the adequacy of the selected processing parameters. The measurement results are presented in Table 5. The relative density of the samples investigated before heat treatment was approximately 99% for both alloys. The results obtained for the heat-treated samples indicate that the heat treatment had no effect on their relative density.

### 3.2. Surface Topography

Table 6 summarizes the average Sa and Sz values, which were used to evaluate the surface roughness of the investigated samples. The analysis was first conducted on the surface roughness of the samples prior to heat treatment. In this case, the average Sa values ranged from 9.7 ± 1.2 µm to 14.4 ± 2.4 µm for samples fabricated from Ti-6Al-4V and from 14.2 ± 0.4 µm to 21.4 ± 1.7 µm for samples fabricated from Ti-6Al-7Nb. The value obtained were higher than those reported in previous studies. These findings support the previously proposed hypothesis that the surface roughness of L-PBF-fabricated components is influenced not only by process parameters but also by additional factors that may be difficult or impossible to control during manufacturing. Moreover, variations in roughness parameters were observed among samples produced using the same strategy, which can be attributed to their position and orientation within the build chamber. This further emphasizes the importance of considering the spatial orientation of individual surfaces when evaluating surface quality.

The measurement results for the heat-treated samples indicate that the treatment had no effect on surface roughness as expressed by the Sa parameter. However, the Sz values decreased. This can be attributed to local plasticization and micro-diffusion of the material, leading to slight smoothing of surface artifacts typical of L-PBF, such as partially melted powder particles, sphericity effects, and laser path irregularities. These changes are subtle and non-uniform, representing a secondary effect rather than the primary objective of heat treatment in L-PBF-fabricated materials.

### 3.3. Microhardness

Table 7 presents the average microhardness values for the five regions of the samples investigated. Due to the rapid heating, melting, and solidification of the alloy, different areas of components produced via L-PBF experience multiple cycles of heating and cooling, which affects their local microstructure and properties. This is likely the main reason for the observed variability in microhardness across the different regions [36,37,38].

**Figure 1:**
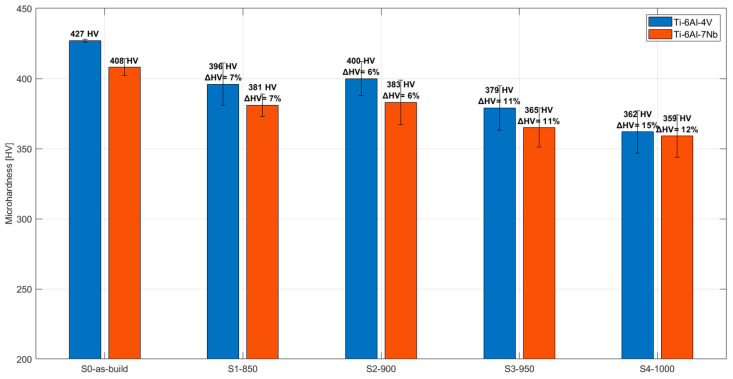
Variation in microhardness of Ti-6Al-4V and Ti-6Al-7Nb alloys with changing heat treatment conditions.

Figure 1 presents the changes in microhardness of the core region of the samples investigated with varying heat treatment conditions. Analysis of the results showed that increasing the annealing temperature was associated with a decrease in microhardness. For Ti-6Al-4V, the microhardness after heat treatment according to the S4_1000 strategy was 362 ± 25 HV, which is 15% lower than in the as-built condition (427 ± 1 HV). In the case of Ti-6Al-7Nb, the decrease in microhardness was 12% (from 408 ± 6 HV in the as-built state to 359 ± 15 HV after heat treatment). The reduction in microhardness reflects changes in the metallographic structure induced by heat treatment. As the annealing temperature increases, the α/α′ → β phase transformation occurs. The β phase exhibits lower hardness, so an increase in its fraction within the microstructure leads to a decrease in the overall microhardness of the alloy [9]. The increased β-phase fraction was also confirmed by other analyses.

### 3.4. Microstructure

**Figure 2:**
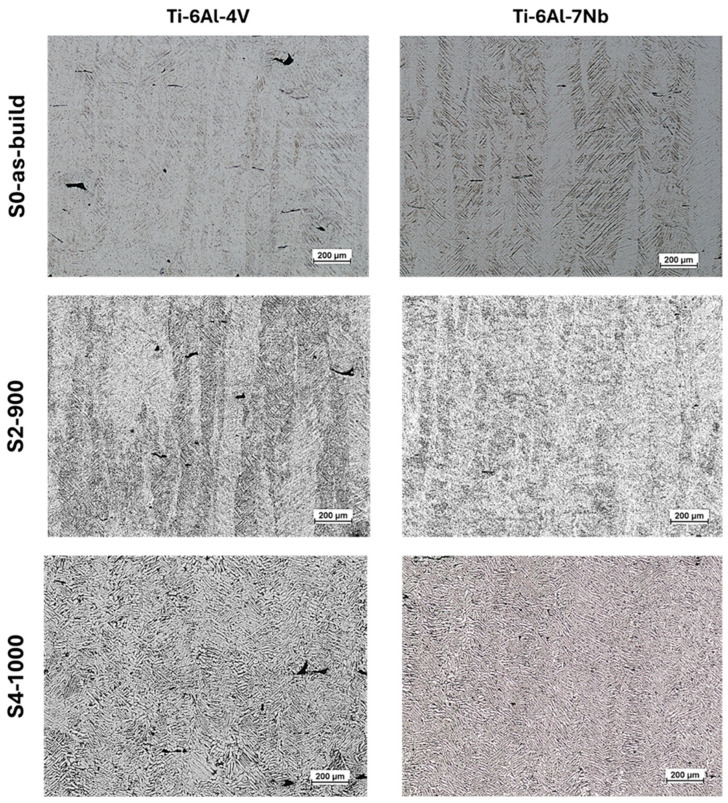
Metallographic structure of Ti-6Al-4V and Ti-6Al-7Nb alloys in the as-built state (S0_as-built) and after heat treatment with annealing below the β-trans temperature (900 °C, S2-900) and above the β-trans temperature (1000 °C, S4-1000).

A material’s metallographic structure largely determines its mechanical behavior. As shown in Figure 2, the as-built microstructure (S0_as-built) of the investigated titanium alloys is characteristic of dual-phase alloys produced via L-PBF [10,11]. Rapid solidification during L-PBF induces the formation of a metastable α′ phase within the columnar β grains, corroborating previous research [16,39].

**Figure 3:**
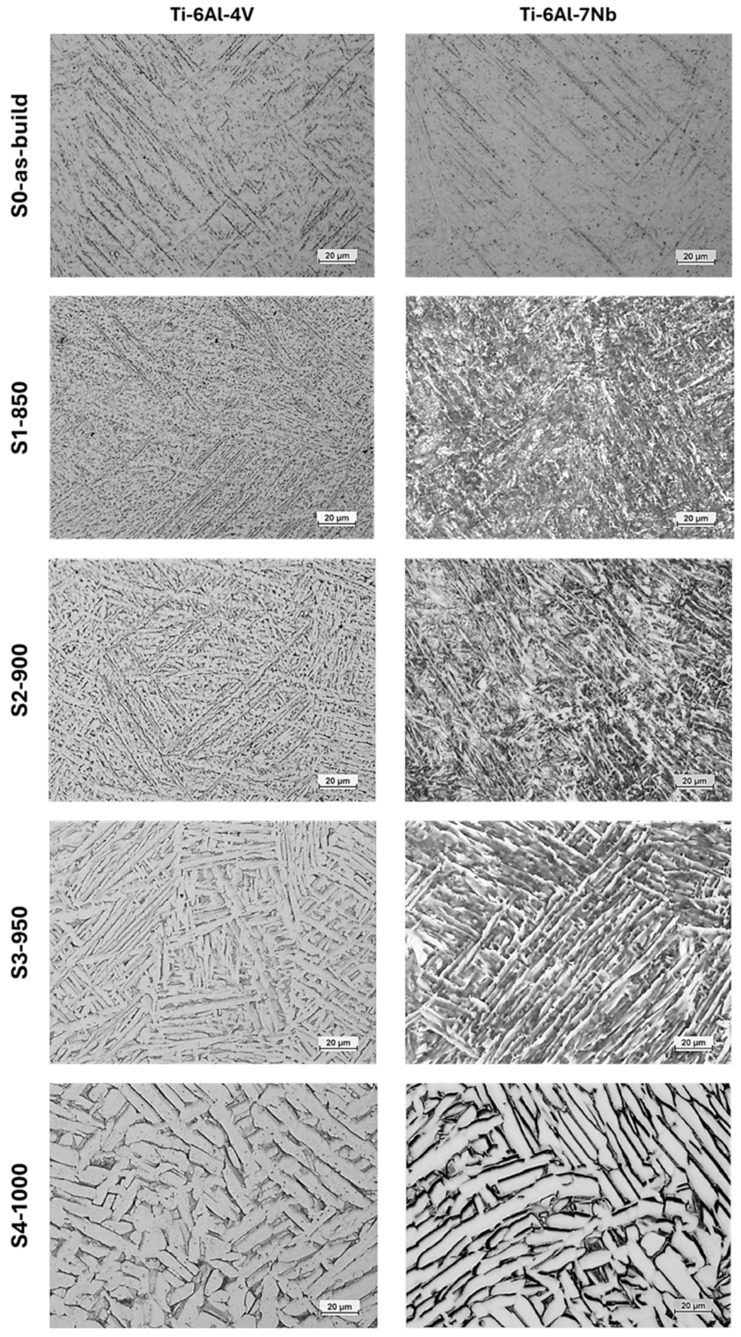
Evolution of the metallographic structure of titanium alloys with varying heat treatment conditions.

**Figure 4:**
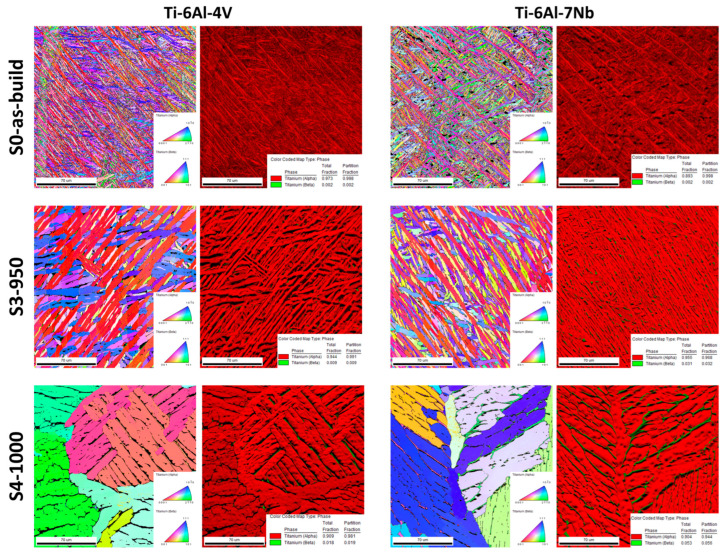
Orientation and phase maps. Coloring based on the fundamental triangle in the direction perpendicular to the map. Reference phases: α-Ti (hexagonal close-packed, HCP) and β-Ti (body-centered cubic, BCC).

Images of the etched metallographic specimens (Figure 3), accompanied by orientation maps and phase maps (Figure 4), enabled a comprehensive assessment of the microstructural evolution of the investigated alloys depending on the heat treatment conditions. As the annealing temperature increases, the primary α′ martensite phase transforms into an α + β phase mixture. Image analysis indicates that both alloys begin to exhibit a Widmanstätten (or basket-weave) microstructure. The acicular α′ phase transforms into the stable α phase, and further increases in annealing temperature result in a pronounced thickening of the α-phase plates. When heat treatment is conducted below the β-trans temperature, the columnar grain structure is preserved (Figure 2, S2-900).

Subjecting both alloys to heat treatment above the β-trans temperature increases the volumetric fraction of the β phase until partially equiaxed grains of this phase are formed, as confirmed by the data presented in Table 8. During slow furnace cooling, these grains transform into a lamellar α + β structure, resulting in a dual-phase microstructure. Ultimately, heat treatment above the β-trans temperature completely transformed the columnar structure into an equiaxed one (Figure 2, S4-1000).

**Figure 5:**
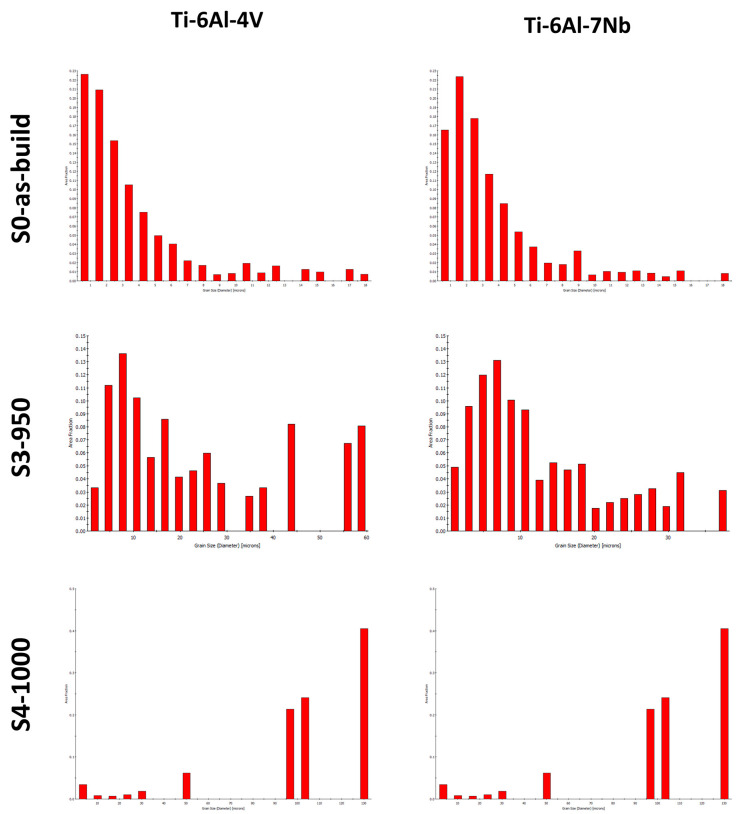
Grain size distribution histograms for the tested alloys.

Figure 5 presents grain size distribution histograms for the investigated samples. Analysis of the histograms revealed that the as-built Ti-6Al-4V and Ti-6Al-7Nb alloys are characterized by a fine-grained microstructure, with grain sizes in range from several nanometers to 18 µm and 19 µm, respectively. Modification of the heat treatment parameters leads to a pronounced increase in grain size: for the S4-950 strategy, the maximum grain size reached 60 µm for Ti-6Al-4V and 48 µm for Ti-6Al-7Nb, whereas under the S5-1000 condition a further increase up to 130 µm was observed for both alloys. The grain growth observed in Ti-6Al-4V and Ti-6Al-7Nb alloys during heat treatment results from several correlated mechanisms. Elevated temperature and soaking time enhance atomic mobility, thereby intensifying diffusion and grain boundary migration and promoting coalescence and recrystallization processes. The α↔β phase transformations increase the fraction of the more mobile β phase, which facilitates the formation of larger grains. In addition, alloying elements such as V and Nb further modify the grain growth kinetics [40,41].

The higher fraction of the β phase observed in the Ti-6Al-7Nb alloy compared to Ti-6Al-4V can be attributed to the stronger β-stabilizing effect of niobium. Nb is an isomorphous β stabilizer that more effectively lowers the β → α transformation temperature in titanium than vanadium [42,43]. As a result, a greater amount of the β phase can be retained at room temperature, even after heat treatment involving aging.

In addition, the diffusion of Nb in the titanium matrix is slower than that of V, which promotes kinetic suppression of the β → α transformation during cooling after aging. This leads to the stabilization of metastable β phase and limits its decomposition into α and α′ phases [44,45].

**Figure 6:**
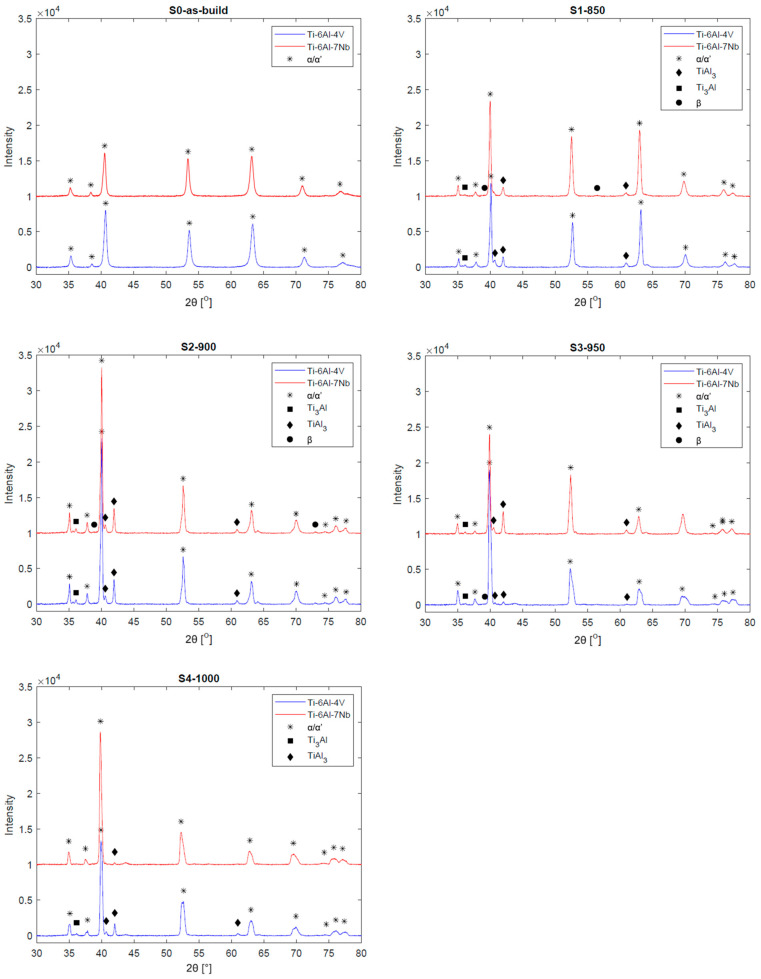
X-ray diffraction spectrums of the investigated titanium alloys before and after heat treatment.

To further assess the phase composition of the investigated samples, X-ray diffraction (XRD) analysis was performed. The obtained diffractograms are presented in Figure 6. In the XRD patterns of the as-built samples (i.e., prior to heat treatment), peaks characteristic of a hexagonal close-packed (HCP) phase are observed. The specifics of the L-PBF process, particularly the high solidification rates of the molten pool, allow these peaks to be attributed to the presence of the metastable α′ phase [17,28,46].

The intensity of the characteristic α′/α peaks in the heat-treated samples was higher compared to the as-built sample, indicating the dissolution of the α′ phase and the growth of α-phase grains. In the XRD patterns of samples subjected to heat treatment below the β-trans temperature, peaks likely originating from intermetallic Ti_3_Al and TiAl_3_ phases were observed. As previously noted, increasing the heat treatment temperature promotes the formation and growth of the β phase. However, the intensity of the characteristic peaks of this phase in the analyzed patterns was low. This is likely due to the high content of the α-phase stabilizer, namely aluminum, in the investigated alloys [47,48].

### 3.5. Mechanical Properties

The microstructure of a material, including titanium alloys, is a key factor strongly influencing its mechanical properties. Table 9 summarizes the parameters describing the mechanical properties of the investigated alloys, including Young’s modulus (E), ultimate tensile strength (Rm), and elongation (A).

**Figure 7:**
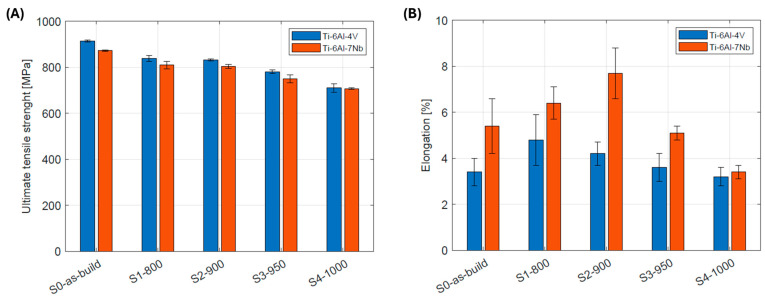
Changes in mechanical properties: (**A**) tensile strength; (**B**) elongation with varying heat treatment conditions for the investigated Ti-6Al-4V and Ti-6Al-7Nb alloys.

The observed changes in the mechanical properties of tested alloys are closely correlated with their microstructural evolution under successive heat treatment strategies. In the as-built state, both materials exhibit a very fine, acicular α′ martensitic microstructure, resulting in the highest tensile strength alongside the lowest elongation (Figure 7). Annealing at 850 –900 °C leads to a gradual decomposition of the martensite and the formation of a fine, equilibrium α + β phase mixture. As a result, a decrease in tensile strength is observed (by 9% for both alloys), accompanied by a significant increase in ductility (41.2% for Ti-6Al-4V and 42.6% for Ti-6Al-7Nb). Further increasing the heat treatment temperature to 950 °C and 1000 °C causes intensive coarsening of the α lamellae and growth of the β grains, leading to reductions in both tensile strength and elongation. The obtained results clearly indicate that the optimum mechanical properties for both alloys are achieved within the 850–900 °C temperature range.

The Ti-6Al-7Nb alloy exhibits higher elongation than Ti-6Al-4V, due to stronger β-phase stabilization by Nb and its higher volumetric fraction. At the same time, this alloy shows slightly lower tensile strength compared to Ti-6Al-4V, due to the reduced α-phase fraction and diminished dispersion strengthening. The maximum elongation of ~7.7% is below the ISO 5832-3 requirement of ≥10% for medical implants applications. This behavior may also be influenced by the specific parameters of L-PBF process, which affect the microstructure and phase distribution.

This indicates that, despite its favorable combination of strength and microstructural stability, the alloy’s ductility may be insufficient for certain load-bearing biomedical applications without further optimization. Heat treatment strategies need to be adapted to account for the unique microstructural characteristics introduced by L-PBF in order to optimize ductility for load-bearing applications. Potential strategies to enhance ductility, such as modifying heat treatment parameters or applying post-processing treatments, could be considered to improve its practical suitability.

## 4. Conclusions

The aim of this study was to evaluate the evolution of the microstructure and mechanical properties of Ti-6Al-4V and Ti-6Al-7Nb titanium alloys under varying heat treatment conditions. The alloys were additively manufactured using L-PBF and subjected to heat treatment consisting of annealing at 850 °C, 900 °C, 950 °C, or 1000 °C for 1 h, followed by aging at 500 °C for 4 h. The process was conducted in a high-purity argon atmosphere with slow furnace cooling.

Based on the experiments and analyses conducted, the following conclusions were drawn:

1. The applied heat treatment had no significant effect on the relative density of the fabricated Ti-6Al-4V and Ti-6Al-7Nb alloys. Heat treatment at temperatures below the melting point did not induce additional discontinuities, such as porosity or microcracks, that could affect the density of the components. The samples retained a similar degree of densification. Therefore, any observed changes in mechanical properties should be primarily attributed to transformations occurring in the metallographic structure of the alloys.
2. The effect of heat treatment on the topography of the upper surface is limited and non-uniform. The observed changes were primarily associated with the partial smoothing of L-PBF-specific surface irregularities due to local plasticization and micro-diffusion of the material, resulting in a reduction in the Sz parameter.
3. The investigated alloys subjected to heat treatment under identical processing conditions exhibited similar metallographic structures. Analysis of etched metallographic images, combined with EBSD and XRD data, clearly indicates that increasing the annealing temperature led to a complete reconstruction of the titanium alloys’ microstructure: from the columnar structure typical of L-PBF to a stable, equilibrium Widmanstätten microstructure characterized by increased homogeneity.
4. The alloys investigated exhibited the same trend in mechanical property changes with varying annealing temperatures, which is closely correlated with the corresponding changes in their metallographic structure.
5. With increasing annealing temperature, a significant decrease in tensile strength was observed for both alloys, which can be attributed to the gradual decomposition of the α/α′ martensite and the formation of a fine, equilibrium α + β phase mixture. The highest elongation values were achieved within the 850–900 °C temperature range. With further increases in heat treatment temperature, the dominant factor limiting the mechanical properties becomes the coarsening of α lamellae and the growth of lamellar colonies.
6. The Ti-6Al-7Nb alloy exhibited higher elongation values compared to Ti-6Al-4V. This difference is attributed to the higher fraction of the β phase in its microstructure, which promotes increased material ductility. This indicates that, despite similarities in the overall metallographic structure of both alloys, their mechanical response is differently influenced by the proportion of the individual phases.

The obtained results represent a significant contribution to the advancement of additive manufacturing technology for titanium alloys, highlighting the critical role of the controlled α′ → α + β transformation and the volumetric fraction of individual phases in determining the functional properties of titanium alloys.

These findings can have a direct impact on the optimization of manufacturing processes for medical implants and structural components used in aerospace applications. In the case of load-bearing implants, the ability to control the β-phase fraction and the intensity of α-lamella coarsening enables the design of structures with enhanced ductility and resistance to brittle fracture. Therefore, heat treatments that maximize β-phase stabilization and ductility may be preferred for medical implants, where elongation and toughness are critical. In the other hand, heat treatments enhancing tensile strength and hardness are better suited for aerospace components, where load-bearing capacity is paramount. Moreover, the experimental data on microstructural evolution provide a solid basis for the development and validation of advanced predictive models, reducing the number of experiments required in the design of new components and streamlining the certification process for additively manufactured parts.

In summary, the obtained results provide a foundation for both the further development of technology and the practical implementation of solutions based on the additive manufacturing of titanium alloys in industries with the highest material performance requirements. Future research should focus on the optimization and tailoring of heat treatment cycles to the specific microstructural characteristics and performance requirements of additively manufactured titanium alloys, enabling further improvement in their functional properties and reliability.

## Figures and Tables

**Table 1:** Chemical composition of the structural material: Ti-6Al-4V and Ti-6Al-7Nb powders (wt. %) [16].

Powder	Ti	Al	V	Nb	Fe	O	C	N	H
**Ti-6Al-4V**	Balance	6.00	4.00	-	≤0.25	≤0.13	≤0.28	≤0.03	≤0.012
**Ti-6Al-7Nb**	Balance	6.05	-	7.1	0.15	0.08	0.015	0.016	0.001

**Table 2:** L-PBF process parameters.

Parameter	Value
Laser power, P [W]	250
Scanning speed, v [mm/s]	1200
Hatch spacing, Hs [mm]	0.1
Layer height, Lh [mm]	0.03
Volumetric laser energy, Ev [J/mm^3^]	70

**Table 3:** Heat treatment strategies.

Strategy Name	Annealing	Aging	Cooling
S1_850	850 °C/1 h	500 °C/4 h	furnace with an argon atmosphere, cooling rate: ~120 °C/1 h
S2_900	900 °C/1 h		
S3_950	950 °C/1 h		
S4_1000	1000 °C/1 h		

**Table 4:** Number of identified grains.

Strategy Name	Ti-6Al-4V	Ti-6Al-7Nb
S0_as_build	33,673	28,669
S3_950	1365	4580
S4_1000	948	968

**Table 5:** Relative density of the tested titanium alloys before and after heat treatment.

Strategy Name	Before Heat Treatment		After Heat Treatment	
	d_AVG_ [g/cm^3^]	d_AVG_ [%]	d_AVG_ [g/cm^3^]	d_AVG_ [%]
**Ti-6Al-4V**				
S1_850	4.39 ± 0.02	99.10	4.39 ± 0.01	99.10
S2_900	4.37 ± 0.01	98.65	4.38 ± 0.01	98.87
S3_950	4.37 ± 0.01	98.65	4.38 ± 0.01	98.87
S4_1000	4.38 ± 0.01	98.87	4.38 ± 0.01	98.87
**Ti-6Al-7Nb**				
S1_850	4.470 ± 0.01	99.11	4.470 ± 0.01	99.11
S2_900	4.480 ± 0.01	99.33	4.480 ± 0.01	99.33
S3_950	4.480 ± 0.01	99.33	4.480 ± 0.01	99.33
S4_1000	4.460 ± 0.01	98.89	4.470 ± 0.02	99.11

**Table 6:** The values of the Sa and Sz parameters for the tested titanium alloys before and after heat treatment.

Strategy Name	Before Heat Treatment		After Heat Treatment	
	Sa [µm]	Sz [µ]	Sa [µm]	Sz [µ]
**Ti-6Al-4V**				
S1_850	13.5 ± 2.4	349 ± 17	13.9 ± 2.4	350 ± 21
S2_900	9.7 ± 1.2	362 ± 96	10.1 ± 1.5	264 ± 71
S3_950	9.7 ± 1.5	351 ± 99	10.4 ± 1.6	285 ± 16
S4_1000	11.0 ± 1.4	398 ± 69	11.6 ± 1.8	338 ± 46
**Ti-6Al-7Nb**				
S1_850	14.8 ± 1.4	433 ± 43	14.9 ± 1.3	357 ± 16
S2_900	14.2 ± 0.4	409 ± 41	14.1 ± 0.1	381 ± 49
S3_950	15.2 ± 1.8	410 ± 39	15.2 ± 2.3	340 ± 17
S4_1000	14.9 ± 2.1	375 ± 24	15.0 ± 3.1	330 ± 48

**Table 7:** Microhardness for the tested titanium alloys before and after heat treatment (OB1—top right corner of the cross-section; OB2—top left corner of the cross-section; OB3—bottom right corner of the cross-section; OB4—bottom left corner of the cross-section; OB5—core).

Strategy Name	OB1	OB2	OB3	OB4	OB5
**Ti-6Al-4V**					
S0_as_build	447 ± 7	444 ± 4	437 ± 14	432 ± 4	427 ±1
S1_850	406 ± 23	424 ±10	395 ± 17	399 ± 9	396 ± 15
S2_900	414 ± 5	391 ± 20	380 ± 15	391 ±6	400 ± 12
S3_950	381 ± 7	390 ± 13	419 ± 12	377 ± 4	379 ± 16
S4_1000	377 ± 5	406 ± 18	368 ± 25	412 ± 39	362 ± 25
**Ti-6Al-7Nb**					
S0_as_build	423 ± 9	413 ± 10	369 ± 5	418 ± 3	408 ± 6
S1_850	413 ± 11	437 ± 17	408 ± 18	409 ± 7	381 ± 8
S2_900	409 ± 17	387 ± 22	385 ± 10	386 ± 6	383 ± 16
S3_950	353 ± 11	369 ± 3	385 ± 4	379 ± 12	365 ± 14
S4_1000	337 ± 10	334 ± 3	362 ± 11	367 ± 9	359 ± 15

**Table 8:** Phase volume fractions of the investigated titanium alloys before and after heat treatment.

Strategy Name	Phase Volume Fractions [%]	
	α-Ti	β-Ti
S0-as-build	99.8	0.2
S3-950	99.1	0.9
S4-1000	98.1	1.9
S0-as-build	99.8	0.2
S3-950	96.8	3.2
S4-1000	94.2	5.6

**Table 9:** Mechanical properties of the titanium alloys investigated before and after heat treatment.

Strategy Name	E [GPa]	Rm [MPa]	ΔRm [%]	A [%]	ΔA [%}
**Ti-6Al-4V**					
S0-as-build	70 ± 4	914 ± 5	----	3.4 ± 0.6	----
S1-850	80 ± 2	838 ± 13	−8	4.2 ± 1.1	23.5
S2-900	78 ± 7	832 ± 5	−9	4.8 ± 0.5	41.2
S3-950	86 ± 5	781 ± 8	−15	3.6 ± 0.6	5.9
S4-1000	84 ± 7	710 ± 18	−22	3.2 ± 0.4	−5.9
**Ti-6Al-7Nb**					
S0-as-build	66 ± 7	872 ± 4	----	5.4 ± 1.2	----
S1-850	80 ± 5	810 ± 16	−7	6.4 ± 0.7	18.5
S2-900	73 ± 4	804 ± 8	−8	7.7 ± 1.1	42.6
S3-950	77 ± 5	749 ± 17	−14	5.1 ± 0.3	−5.6
S4-1000	63 ± 3	707 ± 3	−19	3.4 ± 0.3	−37.0

## Data Availability

The data that support the findings of this study will be made available in RepOD (https://doi.org/10.18150/OVHIGF) upon acceptance of the manuscript.
